# A non-randomized, open-label study of the safety and effectiveness of a novel non-pneumatic compression device (NPCD) for lower limb lymphedema

**DOI:** 10.1038/s41598-022-17225-9

**Published:** 2022-08-17

**Authors:** Stanley G. Rockson, Pinar Karaca-Mandic, Michelle Nguyen, Kristin Shadduck, Phyllis Gingerich, Elizabeth Campione, Heather Hetrrick

**Affiliations:** 1 Cardiovascular Medicine, Falk Cardiovascular Research Center, Stanford University, Stanford, CA 94305 USA; 2 Carlson School of Management, University of Minnesota, Minneapolis, MN USA; 3 PT Works, Palo Alto, CA USA; 4 Ginger-K Cancer Center, Morgan Hill, CA USA; 5 Department of Physical Therapy, Midwestern University, Downers Grove, IL USA; 6 Department of Physical Therapy, Nova Southeastern University, Ft. Lauderdale, FL USA

**Keywords:** Cancer, Quality of life

## Abstract

Lower extremity lymphedema (LEL) can result in detriments to quality of life (QOL) and impose a significant economic burden on patients and payers. A common component of treatment is pneumatic compression, which requires patients to remain immobile. We investigated a novel non-pneumatic compression device (NPCD) that allows patients to remain active during compression treatment, to see if it reduces swelling and improves QOL. We conducted a non-randomized, open-label, 12-week pilot study of adult patients with primary or secondary unilateral LEL, and measured changes in limb edema and QOL using the Lymphedema Quality of Life Questionnaire (LYMQOL). Twenty-four subjects were enrolled; the majority were female (17) with secondary lymphedema (21). Eighteen completed the study. Statistically significant improvements were observed in overall QOL, aggregated LYMQOL total score, and three of four LYMQOL subscales (Function, Appearance, Mood). The fourth (Symptoms) trended toward significant improvement (p = 0.06). The average reduction in affected limb edema was 39.4%. The novel NPCD produced statistically significant improvements in QOL, functioning, and edema volume of patients with LEL. Innovations in devices to manage LEL can be effective while allowing patients to maintain mobility and physical activity during treatment.

## Introduction

Lower extremity lymphedema (LEL) occurs with a reduction in lymph flow that may be caused by trauma, malignancy, infection, or venous/lymphatic disorders^1,2^. While secondary lymphedema is often thought of as a result of cancer treatment, venous disorders have been underrecognized as a cause and may, in fact, be the most common cause of LEL in the US^3,4^. Progression of LEL often results in cellulitis and infections^2–5^, which can further worsen lymphatic drainage that will exacerbate edema^2^. Other skin ailments, such as hyperkeratosis and papillomatosis can also occur^2^.

Significant detriments to physical functioning and quality of life (QOL) are also common. LEL can impair sleep, physical activity, daily activity, housework, social activities, and psychological wellbeing^6–9^, as well as physical and sexual functioning^7,10^. Lymphedema results in significant healthcare costs. It has been estimated that between 2012 and 2017, more than $1B was spent on lymphedema-related hospitalizations; often hospitalizations involving the lower extremities are more costly than those for upper extremities^11^. The physical limitations imposed by LEL have been shown to increase the likelihood of needing assistance with activities of daily living^12^, which can impose significant costs to patients.

Treatment goals include preventing disease progression, maintaining limb size, and avoiding skin infections, and often include compression and exercise^2,13^. Pneumatic compression has been shown to reduce infections and cellulitis and save costs through the avoidance of complications and healthcare encounters^14–17^. A prospective study of LEL patients observed a 32% decrease in cellulitis, 26% reduction in hospitalization, and 24% decrease in the need for physical therapy associated with the use of pneumatic compression^15^. However, most pneumatic compression devices require patients to remain immobile during treatment. Conversely, active exercise, when combined with compression therapy, has been shown to significantly improve volume reduction in LEL^18^.

**Figure 1:**
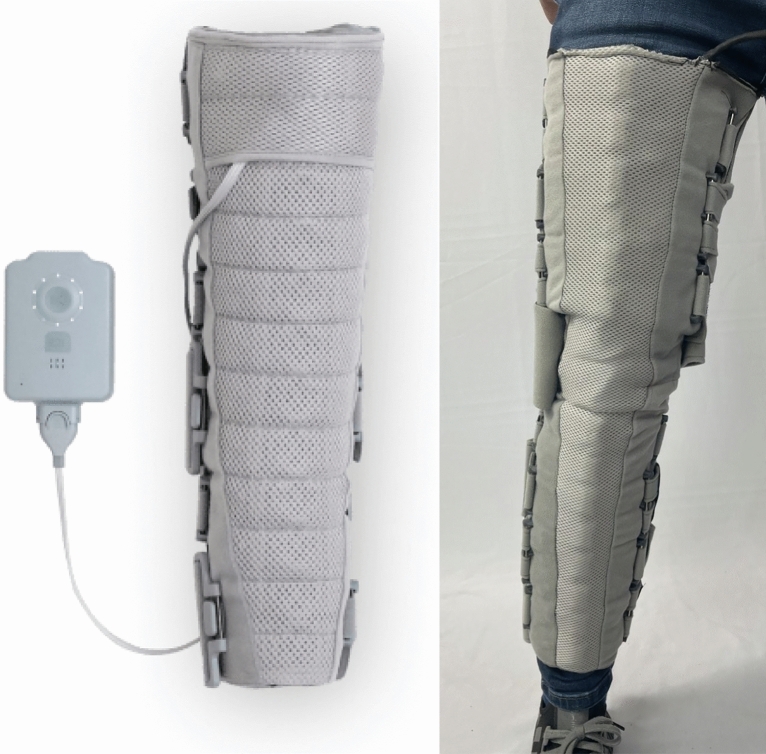
The novel non-pneumatic compression device.

The Dayspring® non-pneumatic compression device (NPCD) is an FDA-cleared prescription-only wearable compression system (Fig. 1) that allows patients to remain active and mobile during compression treatment. It covers the full leg from ankle to hip crease. In this study, we sought to demonstrate that it is safe and effective for use in treatment of LEL. Specifically, we hypothesized that the device maintains or reduces lower extremity swelling and improves QOL.

## Methods

### Study type and patient population

Patients were recruited from two US sites to participate in this non-randomized, open-label, 12-week pilot study. Study methods were developed to comply with accepted guidelines for human subject studies and were reviewed and approved by the Aspire WCG IRB. Prior to a screening visit, patients were provided with IRB-approved informed consent forms and given an opportunity to review and ask questions before offering written consent. The screening visit occurred 1 to 90 days prior to the study start date (“Day 0”). Eligible subjects included all adults (age ≥ 18 years of age) with a diagnosis of primary or secondary unilateral lower extremity edema. If applicable, subjects were required to be at least 3 months post-surgery, chemotherapy, and/or radiation treatment for cancer. Subjects were excluded if they had a prior or current medical condition that might place them at increased risk from sequential compression therapy, or that would prevent safe and effective use of the study device (cellulitis, open-wounds, healing-wounds, etc.). Those with a cognitive or physical impairment that could interfere with the proper use of the device were also excluded. Finally, subjects were excluded if they had participated in any clinical trial of an investigational substance or device during the previous 30 days.

If applicable, 30 days prior to the study start, subjects were asked to cease any current active use of compression device(s) for lymphedema, but to continue to use bandaging, static compression garments and massage, as instructed for their ongoing maintenance care. Subjects were taught how to apply the device themselves, including placement, duration of use, and turning it on/off. All were instructed to wear the device for up to one hour per day and to continue usual activity during use.

### Study measures and outcomes

The primary end-point of QOL was evaluated using the Lymphedema Quality of Life Questionnaire (LYMQOL)^19^, a 20-item validated disease-specific QOL tool, administered at Day 0 and again at study completion. The version of the LYMQOL used in this study specific to the lower extremity and includes an “overall QOL” question (scored 1–10, with a higher score reflecting better QOL), an aggregated total LYMQOL score, and four subscales: Symptoms (pain, swelling, and numbness), Body Image/Appearance, Function (activities of daily living such as eating, writing, and dressing), and Mood (sleep disruption, depression, and irritability). The subscales are scored from 1 (not at all) to 4 (a lot). The aggregated total LYMQOL score is calculated by adding all items and dividing by the total number of items. Within subscales and the aggregated total, lower scores reflect less impairment (i.e., improvement). A co-primary end-point of change in lower limb volume was calculated with tape measure-derived, sequential circumferential measurements of the limb obtained by independently trained Certified Lymphedema Therapists, measured from the ankle to the inguinal crease in 4 cm increments. Limb volume was calculated at Day 0 and at weeks 1, 4, 8, and 12 (study completion). The circumferences were transformed into volumes using a truncated cone approximation and compared to the control (unaffected) limb volume over the duration of treatment. Safety end-points were documented based on any adverse events during the course of the treatment.

### Statistical analyses

Descriptive statistics of QOL, affected lower extremity volume, and percent edema volume reduction included means and standard deviations (SDs) and standard errors (SEs). Changes in LYMQOL scores and mean reduction (as a percent) in edema volume reduction against the unaffected leg from day 0 to week 12 were compared using paired t-tests. P-values less than 0.05 were considered statistically significant.

## Results

A total of 24 subjects were enrolled; 18 completed the study and 6 were lost to follow up. Of the 18 subjects who completed the study events, all responded to the survey at the end of the study. The mean ± SD age was 63.0 (14.0) years, with 17 females and 7 of Caucasian race (Table 1). Both primary (3 patients) and secondary (21 patients) lymphedema were represented; among those with secondary lymphedema, 13 were cancer-related and 8 had chronic venous insufficiency. The patient population included all clinical stages of lymphedema (Stage 1–3) and included subjects that had previously experienced pneumatic compression device treatment.

**Table 1:** Patient characteristics.

Total enrolled	24
Age in years, mean (sd)	63.0 (14.04)
Female (male)	17 (7)
**Race**	
Caucasian	21
African American	2
Hispanic	1
Other	0
**Affected leg**	
Left	13
Right	11

Cell values are counts unless otherwise noted.

**Figure 2:**
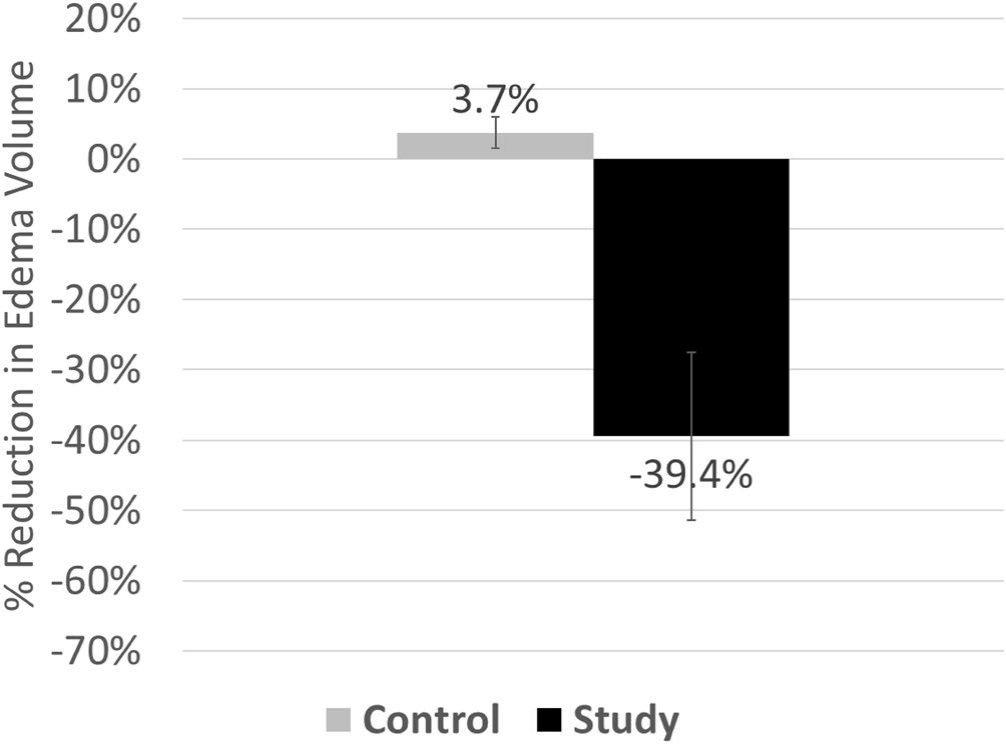
Mean percentage change in edema volume is expressed as the difference in measured volumes of the diseased limb (study) unaffected limb (control), respectively, and expressed as a % change in edema volume (-39.4%, as shown) over 12 weeks. For comparative purposes, the bar graph also contains (in grey) the change in mean volume % of the unaffected limb (control), determined to be 3.7%, as shown, over the same duration.

The primary-end point of QOL showed significant improvement during the course of the treatment. Mean values for the LYMQOL subscales, aggregated total, and “overall QOL” scores improved by 8% to 16% between Day 0 and study completion. Improvements in “overall QOL”, the aggregated total score, and scores for subscales for Function, Appearance, and Mood were statistically significant. The improvement in the subscale Symptoms trended toward significance (p = 0.06, Table 2). The co-primary end-point of limb volume also demonstrated a mean reduction during the course of the treatment. The average edema volume at day 0 was 1271.4 cm^3^; at week 12 the average was 844.3 cm^3^. The mean change in edema was − 427.1 cm^3^ (p < 0.001, 95% confidence interval = − 677, − 178). Edema was reduced by 39.4% from study initiation, on average (Fig. 2). No serious adverse events were reported.

**Table 2:** Change in LYMQOL during study period.

	Mean pre	Mean post	Mean difference	% difference	p-value
Function^a^	1.56	1.39	0.17	11%	0.0003
Appearance^a^	2.14	1.97	0.16	8%	0.01
Symptoms^a^	1.89	1.73	0.16	8%	0.06
Mood^a^	1.47	1.34	0.13	9%	0.02
Overall QOL^b^	7.00	7.83	0.83	12%	0.02

*LYMQOL* Lymphedema Quality of Life Questionnaire, *QOL* quality of life.

^a^A lower score reflects improvement (less impairment).

^b^A higher score reflects improvement.

## Discussion

Treatment for LEL is important to maintain limb size and health and reduce the risk of skin infections^2^. Cellulitis is common among those with LEL, with previous studies reporting occurrences in 30% to 72% of patients, and often with recurrences^3–5^. The use of compression devices can reduce the risk of cellulitis and help to avoid costly healthcare encounters^14–17^ in addition to decreasing edema volume^20^. LEL is associated with significant detriments to QOL^6–9^, and patients express difficulty maintaining physical activity^6,7,9,10^, which is problematic given that physical exercise can increase the effectiveness of compression treatment^18^.

The results of the current study support a statistically significant (although modest) improvement in QOL, including function with use of the novel NPCD. Additionally, limb edema volume significantly decreased during the study. The design of the device allows for movement and patient activity, instead of requiring patients to remain immobile as with many pneumatic compression devices. The average observed reduction in limb edema volume was 39.4%; compared to a previous study of pneumatic compression treatment in LEL, which observed a mean reduction of 8%^20^ that corresponded to improvements in functioning and patient-reported outcomes.

The study includes the use of a validated QOL assessment tool and the inclusion of patients with a variety of causes and stages of LEL. However, a limitation of this study is the lack of a control group, either with use of a comparator pneumatic compression device or comparison with no device use. Control observations would facilitate direct statistical comparison that would strengthen the statistical implications of the observed changes with NPCD use. Additionally, the responsiveness of the LYMQOL to treatment intervention has not been validated, so we cannot determine the clinical significance of the observed domain scores. However, a study of a traditional pneumatic compression device observed no significant change in LYMQOL scores after 12 or 24 weeks^14^; while a significant improvement of 1.1-points was observed after 52 weeks. It is notable that this historical long-term improvement in QOL, as reported, is only modestly higher than the 0.83-point improvement observed in the current study after only 12 weeks of device use. Finally, we did not collect data on adherence; lack of adherence would potentially influence the observed efficacy of the device. Despite these limitations, the observed results across the study subjects over the duration of the treatment produced statistically significant improvements in QOL, functioning, and edema volume. Future studies are needed to directly compare the safety and efficacy of the novel NPCD to a traditional pneumatic compression device for the treatment of LEL.

## Data Availability

The data generated during the currently study are available from the corresponding author upon reasonable request.
