# Author Correction: A non-randomized, open-label study of the safety and effectiveness of a novel non-pneumatic compression device (NPCD) for lower limb lymphedema

**DOI:** 10.1038/s41598-023-28027-y

**Published:** 2023-01-17

**Authors:** Stanley G. Rockson, Pinar Karaca-Mandic, Michelle Nguyen, Kristin Shadduck, Phyllis Gingerich, Elizabeth Campione, Heather Hetrrick

**Affiliations:** 1 Cardiovascular Medicine, Falk Cardiovascular Research Center, Stanford University, Stanford, CA 94305 USA; 2 Carlson School of Management, University of Minnesota, Minneapolis, MN USA; 3 PT Works, Palo Alto, CA USA; 4 Ginger-K Cancer Center, Morgan Hill, CA USA; 5 Department of Physical Therapy, Midwestern University, Downers Grove, IL USA; 6 Department of Physical Therapy, Nova Southeastern University, Ft. Lauderdale, FL USA

Correction to: *Scientific Reports* 10.1038/s41598-022-17225-9, published online 17 August 2022

The original version of this Article contained an error in the Results section, where the overall QOL scores were calculated as the average of means of each patient instead of pooling. As a result,

“Mean values for the LYMQOL subscales, aggregated total, and “overall QOL” scores improved by 8% to 12% between Day 0 and study completion.”

now reads:

“Mean values for the LYMQOL subscales, aggregated total, and “overall QOL” scores improved by 8% to 16% between Day 0 and study completion.”

Additionally, the legend of Figure 2 was corrected to increase the readability of the Figure. As a result,

“Mean percent change in edema from day 0 to week 12 in the study leg versus the control (unaffected) leg. Edema reflects the difference in volume between an individual’s affected leg and unaffected leg. Improvement (i.e., reduction in edema) is presented as the mean percent change in leg edema from day 0 to week 12. A negative percent reflects a reduction in edema.”

now reads:

“Mean percentage change in edema volume is expressed as the difference in measured volumes of the diseased limb (study) unaffected limb (control), respectively, and expressed as a % change in edema volume (-39.4%, as shown) over 12 weeks. For comparative purposes, the bar graph also contains (in grey) the change in mean volume % of the unaffected limb (control), determined to be 3.7%, as shown, over the same duration.”

The original Article has been corrected.

